# Supplementary material for: Chaperones shape the conformational landscape of 26S-proteasome-base assembly for allosteric ATPase motor activation
Source: bioRxiv. 2026 Jun 2:2026.06.01.729410. Preprint. [Version 1] doi: 10.64898/2026.06.01.729410 (PMC13252414; doi:10.64898/2026.06.01.729410)
Supplement: Supplement 1 [file NIHPP2026.06.01.729410v1-supplement-1.pdf]

	**base-Nas2-Rpn14-Hsm3-Nas6**	**base-Rpn14-Hsm3-Nas6**	**base-Rpn14-Hsm3-Nas6(2)**	**base-Hsm3-Nas6**	**Rpn14-Rpt6** (base-Rpn14-Hsm3-Nas6(2))	**Hsm3-Rpt1-Rpt2** (base-Rpn14-Hsm3-Nas6)	**Hsm3-Rpt1-Rpt2** (base-Hsm3-Nas6)	**Hsm3-Rpt1-Rpt2-Rpt3-Rpt4-Rpt5**
EM Data Bank	EMD-77338	EMD-77350	EMD-77358	EMD-77359	EMD-77307	EMD-77304	EMD-77302	EMD-77308
PDB	36AX	36BD	36BL	36BM	35ZV	N/A	35ZR	35ZW
**Data collection and processing**								
Magnification	×81000	×81000	×81000	×81000	×81000	×81000	×81000	×81000
Voltage (kV)	300	300	300	300	300	300	300	300
Electron exposure (e–/Å^2)	50	50	50	50	50	50	50	50
Defocus range (μm)	-0.5 to -2.0	-0.5 to -2.0	-0.5 to -2.0	-0.5 to -2.0	-0.5 to -2.0	-0.5 to -2.0	-0.5 to -2.0	-0.5 to -2.0
Pixel size (Å)	1.048	1.048	1.048	1.048	1.048	1.048	1.048	1.048
Symmetry imposed	C_1	C_1	C_1	C_1	C_1	C_1	C_1	C_1
Initial particle images (no.)	1076993	1076993	1076993	1076993	1076993	1076993	1076993	1076993
Final particle images (no.)	154122	310130	208110	241263	208110	518136	241263	241263
Map resolution (Å)	4.87<br>0.143	3.93<br>0.143	3.91<br>0.143	3.82<br>0.143	3.11<br>0.143	3.08<br>0.143	2.99<br>0.143	3.31<br>0.143
FSC threshold	0.143	0.143	0.143	0.143	0.143	0.143	0.143	0.143
**Refinement**						no model built		
Model resolution (Å)	5.02	3.89	3.96	3.79	3.04		2.95	3.26
FSC threshold	0.143	0.143	0.143	0.143	0.143		0.143	0.143
Map sharpening *B* factor (Å^2)	NA	NA	NA	NA	NA		NA	NA
Model composition								
Non-hydrogen atoms	43182	41629	41530	36449	6397		7786	15764
Protein residues	5472	5280	5266	4617	807		971	1979
Ligands	ATP: 6	ATP: 6	ATP: 6	ATP: 5	ATP: 1		ATP: 2	ATP: 5

B factors (Å^2) (min/max/mean)								
Protein	149.33/1048.92/615.27	79.32/779.92/317.85	88.90/1028.38/366.6	45.22/592.47/221.15	58.46/171.25/102.06		63.19/208.03/108.01	31.60/533.41/154.35
Ligand	247.85/569.79/404.63	201.25/515.45/335.82	189.94/712.02/419.10	124.80/187.07/143.52	91.43/127.62/102.87		82.62/135.43/100.03	47.76/170.84/105.23
R.m.s. deviations								
Bond lengths (Å)	0.004	0.004	0.003	0.003	0.003		0.004	0.003
Bond angles (°)	0.706	0.664	0.659	0.676	0.64		0.664	0.69
Validation								
MolProbity score	2	1.71	1.57	1.75	1.49		1.68	1.59
Clashscore	17.55	11.21	9.29	12	6.83		8.42	9.66
Poor rotamers (%)	0	0.85	0.92	1.27	1.27		1.97	1.05
Ramachandran plot								
Disallowed (%)	0	0.02	0.06	0	0		0	0
Allowed (%)	3.83	2.75	2.3	2.36	2.16		1.77	2.3
Favored (%)	96.17	97.23	97.65	97.64	97.84		98.23	97.7

Supplemental Table 1
